# Riparian Forests and Macroinvertebrates Support Multiple Ecosystem Processes Across Temperate and Tropical Streams

**DOI:** 10.1007/s10021-025-01024-0

**Published:** 2025-11-10

**Authors:** Rebecca Oester, Paula M. de Omena, Larissa Corteletti da Costa, Marcelo S. Moretti, Florian Altermatt, Andreas Bruder

**Affiliations:** 1 Institute of Microbiology, University of Applied Sciences and Arts of Southern Switzerland, Via Flora Ruchat Roncati 15, CH-6850 Mendrisio, Switzerland; 2 Department of Evolutionary Biology and Environmental Studies, University of Zurich, Winterthurerstr. 190, CH-8057 Zurich, Switzerland; 3 Department of Aquatic Ecology, Eawag: Swiss Federal Institute of Aquatic Science and Technology, Überlandstrasse 133, CH-8600 Dübendorf, Switzerland; 4 Laboratory of Aquatic Insect Ecology, University of Vila Velha, Av. Comissário José Dantas de Melo 21, Vila Velha, ES 29102-920 Brazil

**Keywords:** Multifunctionality, Biodiversity, Cross-ecosystem flows, Nutrient cycling, Leaf litter decomposition, Aquatic-terrestrial linkages, Detrital food web

## Abstract

Ecosystems are interconnected, and ecological processes frequently transcend the physical boundaries that define them. Fluxes of energy, matter, and organisms not only form important ecosystem processes within but also between ecosystems. However, the role of biological drivers in simultaneously supporting multiple ecosystem processes at the interface between aquatic and terrestrial ecosystems (that is, aquatic-terrestrial ecosystem processes) remains poorly understood, both locally and across regions. To assess the relative importance of riparian forests, detritus consumers and leaf litter mixing on different ecosystem processes of freshwater detrital food webs, we used leaf litter bags to subsidise local terrestrial leaf litter to forested and non-forested headwater stream sites in a temperate and tropical region. We also manipulated macroinvertebrate access and the composition of leaf litter mixtures. We measured three key aquatic-terrestrial ecosystem processes: biomass accrual of aquatic fungi, nitrogen loss, and decomposition rates of local leaf litter. Across both temperate and tropical streams, ecosystem multifunctionality, that is, the simultaneous sustaining of these processes, was positively associated with macroinvertebrates and riparian forests but not with leaf litter mixing. Especially leaf litter nitrogen loss and decomposition rates were consistently higher when macroinvertebrates had access across all leaf litter species. Decomposition rates were also positively associated with the other ecosystem processes. These findings highlight consistent, cross-regional effects of riparian forests and macroinvertebrate detritivores on freshwater detrital food webs. In a rapidly changing world, understanding ecosystem processes in headwater streams demands a holistic view that transcends ecosystem borders and incorporates cross-ecosystem interactions.

**Supplementary Information:**

The online version contains supplementary material available at 10.1007/s10021-025-01024-0.

## Highlights

- Riparian forests support multifunctionality in temperate and tropical streams.
- Macroinvertebrates and riparian forests enhance multiple ecosystem processes.
- Temperate and tropical streams share biological drivers of ecosystem processes.

## Introduction

Aquatic and terrestrial ecosystems are not isolated but connected by the exchange of energy, matter, and organisms across their boundaries (Baxter and others 2005; Gounand and others 2018; Scherer-Lorenzen and others 2022; Harvey and others 2023). These cross-ecosystem fluxes and subsidies underpin strong ecological linkages and key ecosystem processes (Naiman and others 1993; Barnes and others 2018; Tolkkinen and others 2020). Ecological subsidies thus support complex food webs that combine elements of both donor and recipient ecosystems (Naiman and Décamps 1997; Baxter and others 2005; Scherer-Lorenzen and others 2022). For example, many aquatic consumers feed on terrestrial-derived resources (Ferreira and others 2023). These linkages are central to major ecological frameworks including the river-continuum concept (Vannote and others 1980) and meta-ecosystem theory (Gounand and others 2018; Harvey and others 2023) and are supported by extensive empirical studies (Webster and Meyer 1997; England and Rosemond 2004; Rezende and others 2019).

One of the most important linkages between terrestrial and aquatic ecosystems is the input of terrestrial leaf litter, which fuels freshwater food webs via detrital pathways (England and Rosemond 2004; Baxter and others 2005; Harvey and others 2023; Oester and others 2025). This allochthonous leaf litter is the primary resource for decomposers and detritivores in most forested streams (Cummins and others 1989; England and Rosemond 2004; Wantzen and Wagner 2006; Handa and others 2014; Oester and others 2024). As leaves from the riparian vegetation enter streams, microbes, such as aquatic fungi, rapidly colonise them, increasing their palatability for detritivores (Gessner and Chauvet 1997). Microbial activity thereby modifies leaf litter stoichiometry by nutrient immobilisation or mineralisation (García-Palacios and others 2017). Which of these processes dominates during decomposition influences the direction and magnitude of nutrient dynamics (Finzi and Canham 1998; Webster and others 2009).

**Figure 1:**
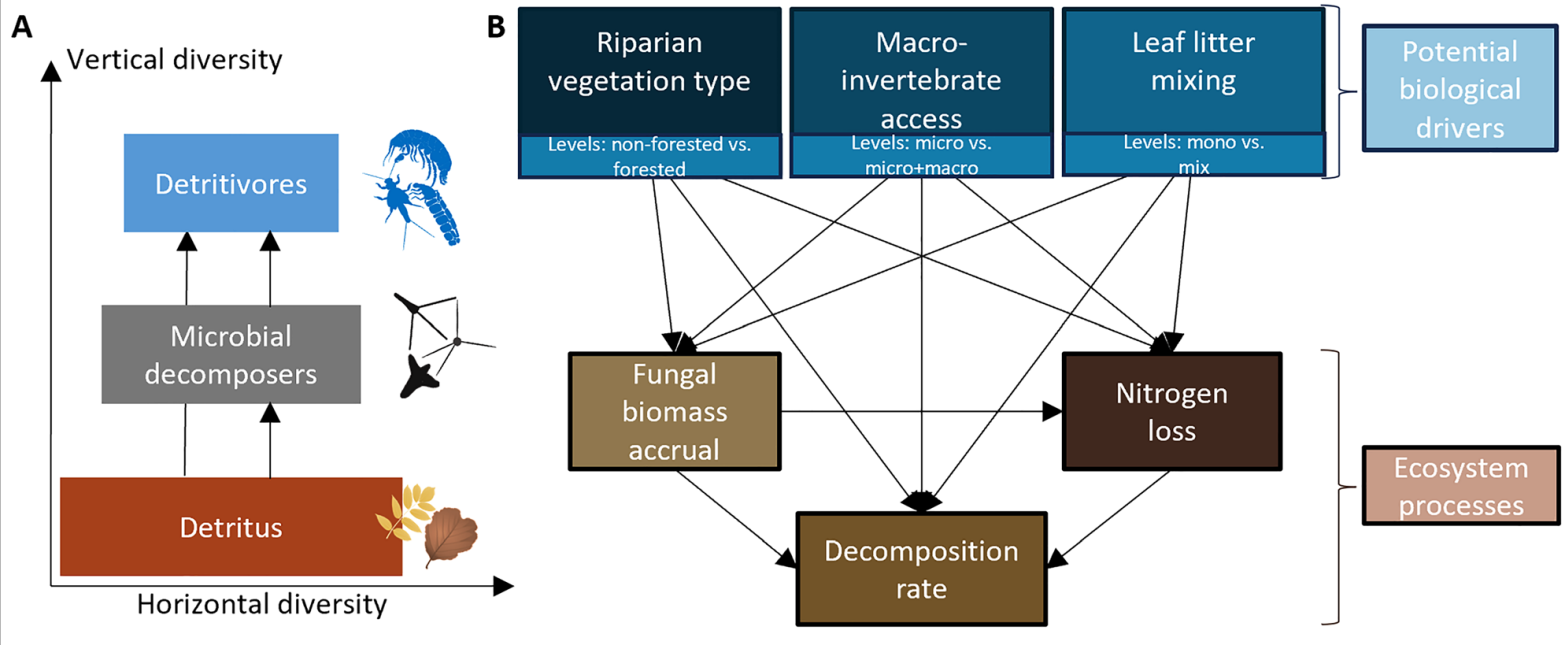
Conceptual figure of detrital food webs in headwater streams and links influencing ecosystem processes. **A** Detrital food web components represented in horizontal diversity (biodiversity within trophic guild) and vertical diversity (biodiversity between trophic guilds; modified from Gessner and others 2010). **B** Metamodel on the direct and indirect effects of potential biological drivers (blue boxes) to individual ecosystem processes (brown boxes). Effects of biological drivers are contrasting non-forested vs. forested vegetation; micro vs. micro + macro community; and mono vs. mixed leaf species on aquatic-terrestrial ecosystem processes in headwater streams represented as path diagram. Detailed hypotheses and literature references for each link can be found in Table S1.

Key ecosystem processes in freshwater detrital food webs combine aquatic and terrestrial components such as in-stream decomposition of terrestrial leaf litter, nutrient dynamics of this detritus, and subsequent transformation into biomass of aquatic consumers (Gessner and Chauvet 1997; Dodds and others 2004; Dang and others 2005; Duarte and others 2006). To disentangle microbial and macroinvertebrate contributions to these aquatic-terrestrial processes, many studies have used leaf litter bags of different mesh sizes, which allow or exclude macroinvertebrates (Cummins and others 1989; Bruder and others 2014; Casotti and others 2015; Omoniyi and others 2021). In addition, leaf litter traits (for example, nutrients, toughness, lignin content) shape decomposition dynamics (Dodds and others 2004; Schindler and Gessner 2009; López-Rojo and others 2019; Santonja and others 2020). Mixing different leaf litter species can provide complementary resources (Moretti and others 2007; Bruder and others 2014; Liu and others 2020). Hence, a greater variety of both resources (horizontal biodiversity) and consumer guilds (vertical biodiversity) can mutually enhance ecosystem processes (Figure 1A; Jonsson and Malmqvist 2000; Gessner and others 2010; Kominoski and others 2010; Jabiol and others 2013; Santonja and others 2020).

Changes in land cover increasingly disrupt the natural flow of terrestrial resources into freshwater ecosystems (Little and Altermatt 2018; López-Rojo and others 2019; Boyero and others 2021). In particular, degradation of riparian forests can alter light and temperature regimes (Naiman and Décamps 1997; Tolkkinen and others 2020; Ferreira and others 2023) and modify the quantity, diversity, and composition of leaf litter inputs (Ohler and others 2023). A recent meta-analysis has demonstrated that these degradations, driven by anthropogenic changes in the watershed and riparian vegetation, occur globally and can negatively impact multiple trophic levels (Oester and others 2025). Together, these disruptions highlight how human impacts can cascade across ecosystem boundaries (Handa and others 2014; Oester and others 2024). Yet, despite decades of work on detrital dynamics, we still lack an integrated understanding of how multiple ecosystem processes (i.e., multifunctionality) are simultaneously sustained - and affected – in headwater streams. Building on a growing body of research linking biodiversity to ecosystem functioning (Lefcheck and others 2015; Soliveres and others 2016), the ability of ecosystems to support multiple processes simultaneously appears to depend on a greater diversity of taxa and trophic levels than single processes (Soliveres and others 2016). Furthermore, uncertainty about the generality of multifunctionality drivers across biomes continues to limit our ability to predict and conserve ecosystem processes in nature and under realistic conditions (Wantzen and Wagner 2006; van der Plas 2019). Hence, understanding how allochthonous inputs support detrital food webs (Beiser and others 1991; Jabiol and others 2013; Barnes and others. 2018) and multiple ecosystem processes (Lefcheck and others 2015; Soliveres and others 2016; Ferreira and others 2023) remains an important research need.

**Figure 2:**
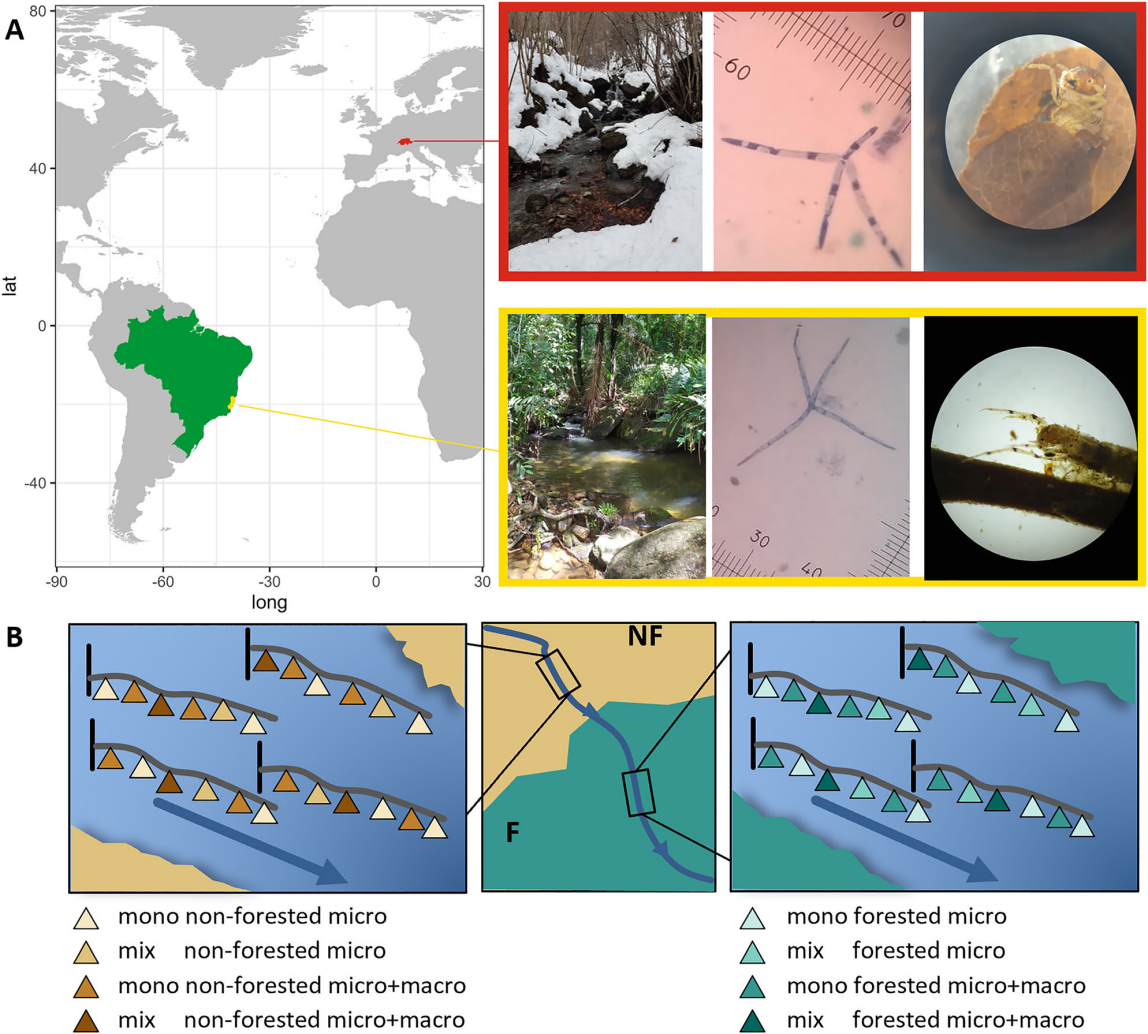
Sites, biodiversity and experimental design. **A** Map of the temperate and tropical study regions: Switzerland is shown in red and Brazil in green, with the state of Espírito Santo highlighted in yellow. Field photos depict study sites, stained conidia of aquatic fungi *Articulospora tetracladia* and *Lemmoniera aquatica* (photos: J. Jabiol), and detritivorous caddisflies (Limnephilidae and Leptoceridae) from Switzerland and Brazil, respectively. **B** Schematic of the experimental design for one of 16 streams surrounded by forested (F) and non-forested (NF) vegetation. Each site received mesh bags containing leaf litter from local tree species—either from a single species (mono) or a mixture (mix). The trees were categorised as nitrogen-fixing (*Alnus glutinosa* in Switzerland, *Inga laurina* in Brazil) or carbon labile (*Fraxinus excelsior* in Switzerland, *Miconia chartacea* in Brazil). Fine mesh bags allowed only microbial decomposition (micro), while coarse mesh bags permitted both microbial and macroinvertebrate access (micro + macro).

To assess key biological drivers of aquatic-terrestrial ecosystem processes (that is, processes occurring at the interface and combining both components of aquatic and terrestrial environments), we examined the roles of riparian forests, macroinvertebrates, and leaf litter mixing. We tested how these factors influence multiple processes, individually and in combination (that is, multifunctionality), in detritus-based food webs of temperate and tropical headwater streams. We used leaf litter bags placed in forested and non-forested stream sites, with mesh that either excluded or allowed access of macroinvertebrates, and filled with either single- or mixed-species leaf litter of common riparian tree species (Figure 1B). We quantified biomass accrual of aquatic fungi growing on leaf litter, nutrient dynamics measured as in-stream nitrogen loss from leaf litter, and aquatic leaf litter decomposition rates (Dang and others 2005; Duarte and others 2006). We replicated these field experiments in headwater streams in Switzerland and Brazil (Figure 2), two regions with distinct climates (Shah et al. 2017), detritus quality (Boyero and others 2017), and biodiversity (Wantzen and Wagner 2006; Boyero and others 2021). While we predicted overall positive effects of riparian forests, macroinvertebrates, and leaf litter mixing on ecosystem processes, we also expected that the relative importance of these biological drivers differed between temperate and tropical regions (Handa and others 2014; Soliveres and others 2016; Boyero and others 2021) as demonstrated in other cross-biome comparisons of forested stream food webs (Wantzen and Wagner 2006; Bruder and others 2014). We expected relatively strong effects of macroinvertebrates as key detritivores (Casotti and others 2015; Oester and others 2023) and litter species-specific mixing effects, due to differences in leaf litter traits and chemical compositions (Schindler and Gessner 2009; Handa and others 2014; Frainer and others 2015). More detailed hypotheses based on our metamodel (Figure 1B) are described in Table S1.

## Material and Methods

### Experimental Design

To test the local effects of riparian forests on aquatic-terrestrial ecosystem processes in temperate and tropical regions, we ran standardised leaf litter decomposition experiments in headwater streams in Switzerland and Brazil (Atlantic Forest biome in the state of Espirito Santo), always contrasting forested and non-forested stream sections (Figure 2A). We selected eight streams in each country, each having a distinct section with the riparian vegetation consisting of densely standing trees (forested) of at least 5 m width on both sides and another section surrounded by locally typical grassland or extensively used pastures with no or only isolated trees (non-forested) (Figure 2B). The river flow length between each two sites within the stream was on average around 500 m. This contrast of forested versus non-forested stream sections is a commonly found landscape pattern of the studied stream systems (Casotti and others 2015; Little and Altermatt 2018; Cereghetti and Altermatt 2023; Ferraz and others 2025). The forested site was upstream in half of the streams and downstream in the other half, which resulted in a balanced and paired comparison between forested and non-forested sites (Oester and others 2023, 2024). The sites were located at elevation levels where the natural riparian vegetation would consist of deciduous forests. Other than the forest–non-forest transition, there were only minimal anthropogenic disturbances or modifications; thus, we measured similar nutrient and temperature levels within the streams (for details on stream and riparian zone characteristics see Table S2 and Oester and others 2023; Ferraz and others 2025).

To test the effects of macroinvertebrate detritivores, we deployed leaf litter bags with different mesh sizes in each site (Cummins and others 1989; Wantzen and Wagner 2006; Oester and others 2023). We used fine and coarse mesh bags (0.25 mm and 10 mm opening, respectively) to exclude or allow the access of macroinvertebrates, respectively. Both types of bags allowed for microbial colonisation of the leaf litter. Hence, the trophic structure within each leaf litter bag consisted of either one (only microbial decomposers) or two (microbial decomposers and macroinvertebrate detritivores) trophic levels, allowing the comparison of food webs with varying detritus consumer levels (micro vs. micro + macro).

At each site, we tested the effects of leaf litter mixing by placing mesh bags with 5 g (SD = 0.03 g) of naturally senesced, dried leaf litter from one or two local tree species. Each bag contained either a single litter species (mono) or a mix (mixed in equal mass). For mixed leaf litter bags, we analysed each species separately. In Switzerland, we used *Alnus glutinosa* (Betulaceae; N-fixing) and *Fraxinus excelsior* (Oleaceae; C-labile), and in Brazil, *Inga laurina* (Fabaceae; N-fixing) and *Miconia chartacea* (Melastomataceae; C-labile). These four tree species are locally common riparian species, with relatively high to moderate nutritional quality and palatability for detritus consumers (Schindler and Gessner 2009; Bruder and others 2014; Kiffer and others 2018). Details on the initial leaf litter characteristics can be found in Table S3 and Figure S2.

Our experimental design allowed us to assess the individual effects of riparian vegetation type (forested vs. non-forested), macroinvertebrate access (micro vs. micro + macro), and leaf litter mixing (mono vs. mix) on ecosystem processes. Each treatment combination was replicated four times, resulting in a total of 24 leaf litter bags per site (768 bags in total). Depending on site conditions (for example, stream width and depth) the leaf litter bags were placed within a 30–50 m reach. We started the field experiments during the local leaf fall season and terminated them when approximately 50% of the initial leaf litter mass was lost. For each stream, all leaf litter bags were collected simultaneously. Consequently, the duration and timing of the field experiment varied between regions, resulting in three to five weeks from December 2020 to January 2021 in Switzerland and six to eight weeks from May to July 2021 in Brazil.

### Ecosystem Processes

To assess aquatic-terrestrial ecosystem processes in freshwater detrital food webs, we examined three ecosystem processes that are key in detritus-based streams that is, fungal biomass accrual (net biomass accumulation of aquatic fungi during the experiment), N loss (% N lost from the leaf litter during the experiment), and leaf decomposition rates (*k* during the experiment) for each leaf litter species (either a single species or two species) in each leaf litter bag resulting in 1024 units in total. We excluded data from leaf litter bags with < 25% leaf litter mass remaining (n = 5, that is, 1.3%), as consumer colonisation and activity might be reduced (Beiser and others 1991).

To determine biomass accrual of aquatic fungi, we quantified net fungal biomass accumulation in the leaf litter at the end of the experiment, assuming no biomass of aquatic fungi in the leaf litter at the beginning of the experiment (Gessner and Chauvet 1997; Grossart and others 2019). We quantified ergosterol, a fungal cell membrane compound, from ten leaf discs cut from different leaves per leaf litter bag and leaf species. After subsampling, we stored the leaf discs at − 20 °C, then freeze-dried them and extracted and purified ergosterol with solid-phase extraction (Sep-Pak® Vac RC tC18 500 mg sorbent; Waters, Milford, USA; Gessner 2020). We then measured the ergosterol concentration using ultra-high-performance liquid chromatography (UHPLC; 1250 Infinity Series, Agilent Technologies, Santa Clara, USA) at a wavelength of 282 nm and estimated net fungal biomass in mg per g leaf litter with a conversion factor of 5.5 mg ergosterol per g fungal biomass (Gessner 2020).

To quantify the detrital nutrient dynamics, we measured the N content of the leaves from dried (60 °C for 48 h), homogenised, and encapsulated (9 × 5 mm tin capsules, Säntis Analytical) leaf litter material (1–1.5 mg) on an Elemental Analyzer (Elementar, Vario Pyro). We calculated the nitrogen (N) loss (%) according to Handa and others (2014): 100 × [(*M*_*i*_ × *N*_*i*_) − (*M*_*f*_ × *N*_*f*_)] / (*M*_*i*_ × *N*_*i*_), where *M*_*i*_ and *M*_*f*_ are the initial and final leaf litter dry mass, respectively, and *N*_*i*_ and *N*_*f*_ are the initial and final N concentration (% of leaf litter dry mass), respectively. Initial *N*_*i*_ was quantified from a representative subset of leaves from the original batch. This calculation reflects the relative loss of N over the experimental time with negative values representing N accumulation due to immobilisation and positive values reflect the dominance of mineralisation over immobilisation since N is only net released (mineralisation > immobilisation) in the later stages of leaf decay (García-Palacios and others 2017).

We quantified decomposition rates using the exponential decay model: *m*_*t*_ = *m*_*0*_* e*^*−kt*^ where *m*_*t*_ is the leaf litter dry weight after *t* degree days, *m*_*0*_ is the initial dry weight, and *k* is the decomposition rate (Webster and Benfield 1986). The decomposition coefficient *k* was calculated by dividing the natural logarithm of the remaining fraction of leaf litter by degree days (Gessner and Peeters 2020). We calculated degree days based on hourly measurements of stream temperature (HOBO Temperature DataLogger; UA-002-64; Onset Computer Corporation) to account for temperature-dependent consumer activity (Wantzen and Wagner 2006; Bruder and others 2014; Ferreira and others 2019).

### Statistical Analysis

To assess multifunctionality, we combined multiple ecosystem processes and calculated an aggregated multifunctional score (Delgado-Baquerizo and others 2016). Multifunctionality refers to a concept that encompasses the quantification of several ecosystem processes provided simultaneously, rather than focusing on a single measurable process. Although some processes were correlated (examined in Supplementary Material Figure S3, Table S5), we treated each process as providing distinct und unique ecological information, following the approach of previous studies in detritus-based streams (López-Rojo and others 2019). To obtain a quantitative multifunctionality index for each leaf species and treatment combination, we first log-transform the values of the ecosystem processes when needed (*k* and fungal biomass accrual). Secondly, we standardised each of the three ecosystem processes to range from 0 and 1 and, thirdly, averaged these standardised ecosystem processes to obtain a single multifunctionality index with the *getStdAndMeanFunctions* function of the multifunc package (Byrnes 2022). This approach and index are commonly employed in multifunctionality research and offer a simple, easily understandable way to assess the capacity to maintain multiple processes simultaneously (Lefcheck and others 2015). The values of multifunctionality range from 0 and 1, and the highest scores are achieved if leaves show high fungal biomass accrual (Gessner and Chauvet 1997), high N loss (Handa and others 2014; García-Palacios and others 2017), and high decomposition rates (Dang and others 2005).

**Figure 3:**
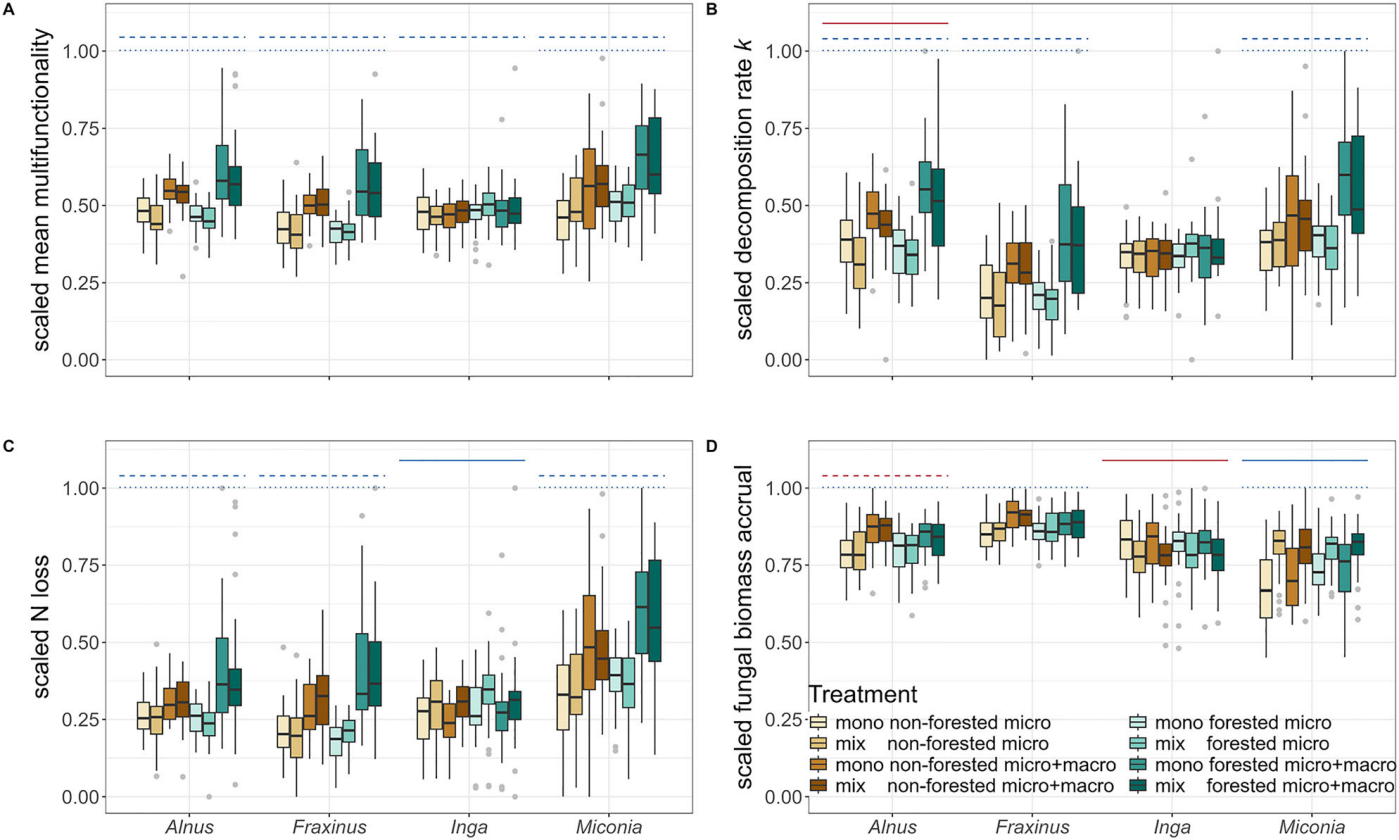
Boxplots of scaled ecosystem processes. **A** mean multifunctionality depending on leaf species and experimental treatment, **B** decomposition rates *k*, **C** nitrogen loss, and **D** fungal biomass. Colours differentiate the treatment combinations of riparian forest and leaf litter mixing and macroinvertebrates, with placement in a forested or non-forested stream section, including mixed (mix) or single (mono) species in leaf litter bags and inclusion (micro + macro) or exclusion (micro) of macroinvertebrate detritivores. Solid lines indicate the effect (CIs not including 0) of the leaf litter mixing, dashed lines indicate the effects of riparian vegetation type (forested vs. non-forested), and dotted lines show the effects of leaf litter mixing (micro vs. micro + macro) separately assessed for each leaf species. Blue lines indicate a positive effect, whereas red lines indicate a negative effect corresponding to the model estimates (Table S4). See Figure S1 and Table S7 for the unscaled and untransformed values.

As the averaging of processes inherently leads to a simplification, has some statistical limitations, and can suffer from biases in defining the process maxima and minima (Meyer and others 2018), we also reported results for each process individually in additional analyses (Figure 3B–D; Figure S1, Table S4, S5). We used the mean multifunctionality score and each ecosystem process as a response variable and tested the effects of riparian forest, macroinvertebrate access and leaf litter mixing separately for the different leaf species in Bayesian generalised nonlinear multivariate multilevel models (brms; Bürkner and others 2021), which use the probabilistic programming language for statistical interference Stan (Stan Development Team 2023). As the multifunctionality score and scaled ecosystem processes are constrained to 0 and 1, we chose weakly informative priors of normally distributed intercepts and slopes (0, 0.5) reflecting these upper and lower bounds and a beta likelihood. To account for the study design and environmental differences among streams, we incorporated sampling location as random effects of the intercept. For all models, we generated 4,000 (four chains run for 2,000 iterations discarding the first 1,000 as burn-in) Markov chain Monte Carlo (MCMC) samples from the posterior distribution where draws were sampled using NUTS (No-U-Turn Sampler).

To assess the direct and indirect effects of riparian forest, leaf litter mixing and macroinvertebrate access, on fungal biomass accrual, N loss and leaf litter decomposition rates, we constructed path diagrams as structural equation models (SEMs) again using Bayesian generalised nonlinear multivariate multilevel models (brms; Bürkner and others 2021). Based on our hypotheses (Table S1) and the metamodel (Figure 1B), we used the same model structure but modelled each leaf species separately to assess the species-specific effects on ecosystem processes. We z-transformed all numeric values and included weakly informative priors of normally distributed intercepts (0, 1) and slopes (0, 0.5). To account for the study design and environmental differences among streams, we again incorporated sampling location as random effects of the intercept with stream nested in region. For all models, we generated 20,000 (four chains run for 10,000 iterations, discarding the first 5,000 as burn-in) MCMC samples from the posterior distribution where draws were sampled using NUTS.

We assessed the model fit with posterior predictive checks and ensured the model diagnostics represent convergence and that the models were well-explored. We visually checked the fit of the posterior distribution with the data (bayesplot, Gabry and others 2021). For all models, MCMC chains indicated convergence by falling within the threshold specified by Gelman and Rubin (1992) and showing high effective sample size measures. When 95% credible intervals of the posterior distribution do not include zero, we refer to these as directional effects.

We performed all calculations and statistical analyses in R (version 4.1.2; R Core Team, 2020). The data and code that support and allow to fully reproduce the findings of this study are openly available on Dryad and Zenodo: 10.5061/dryad.r7sqv9smr; 10.5281/zenodo.13330062.

## Results

The presence of local riparian forests and macroinvertebrate detritivores had predominantly positive effects on combined (Figure 3A) and individual (Figure 3B–D) stream ecosystem processes in temperate and tropical streams. In both temperate and tropical leaf species, scaled mean multifunctionality was higher in forested compared to non-forested sites (*Alnus*: estimate = 0.13, CIs = [0.05, 0.21]; *Fraxinus*: 0.13[0.05, 0.21]; *Inga*: 0.08[0.01, 0.14]; *Miconia*: 0.19[0.09, 0.29]). Moreover, for *Alnus*, *Fraxinus,* and *Miconia*, there were higher multifunctionality scores with the inclusion of macroinvertebrates, (*Alnus*: 0.43[0.35, 0.51]; *Fraxinus*: 0.48[0.39, 0.56]; *Miconia*: 0.52[0.41, 0.62) but not for *Inga* 0.00[− 0.06, 0.07]). Across all leaf species, multifunctionality scores consistently showed no influence of leaf litter mixing (*Alnus*: − 0.07[− 0.15, 0.01]; *Fraxinus*: <  − 0.01 [− 0.08, 0.08]; *Inga*: 0.04[− 0.01, 0.11]; *Miconia*: 0.08[− 0.02, 0.19]).

**Figure 4:**
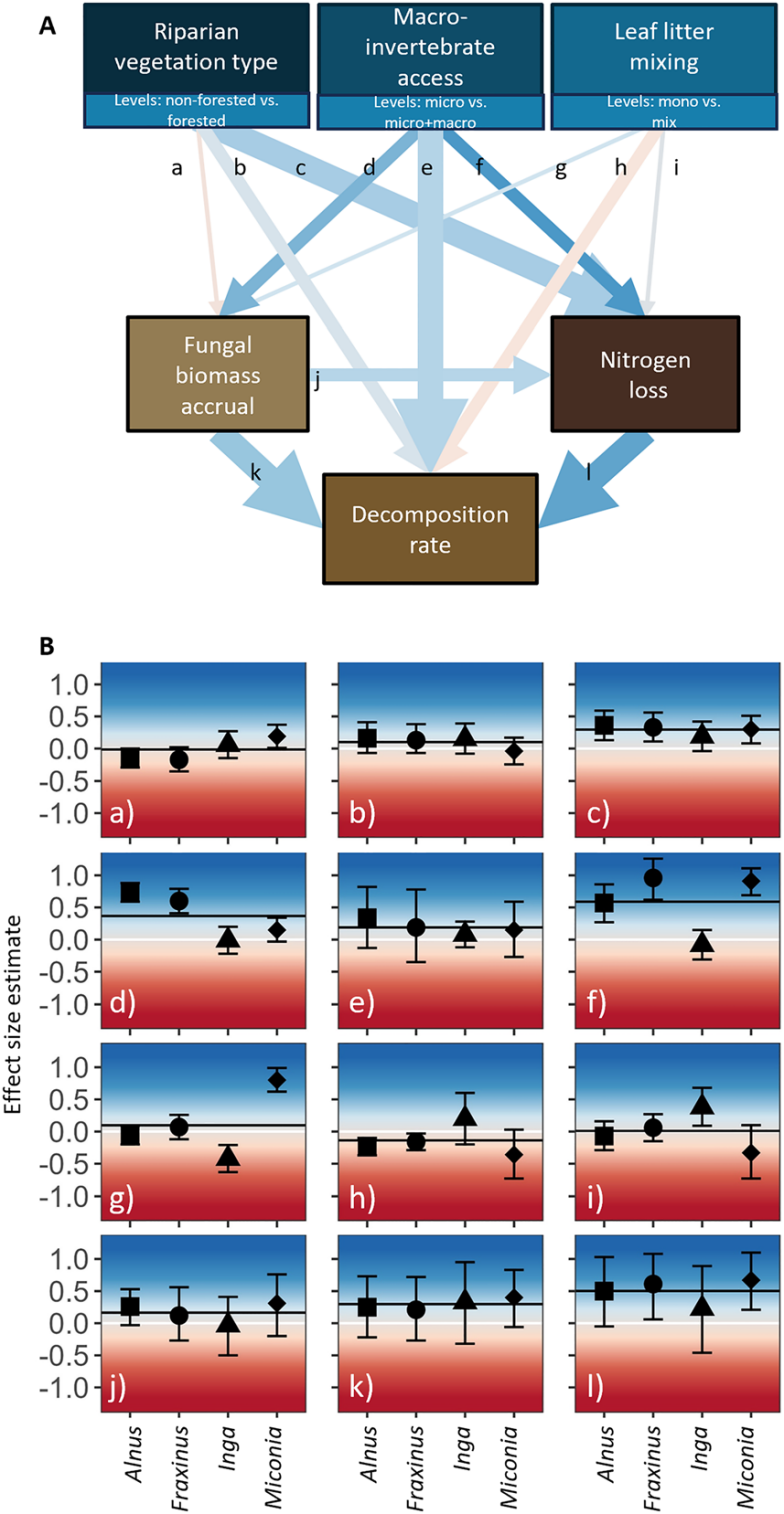
Path model (SEM) and its effect sizes linking biological drivers (blue boxes) to individual ecosystem processes (brown boxes). **A** Effects of biological drivers are always contrasting non-forested vs. forested vegetation; micro vs. micro + macro community; and mono vs. mixed leaf species. For example, if the value of the ecosystem processes for mono is lower than the corresponding mixed value, the effect size estimate is positive for this treatment comparison. The colour of the arrows displays the average effect size ranging from blue (highly positive) to red (highly negative) consistent with the colour palette in B. Thick arrows show consistency in effect direction for all four leaf species, and normal arrows show that three of the four leaf species show consistent directionality, while fine arrows show inconsistent directionality. **B** Model estimates (mean ± CI) of the SEMs for each leaf species and separated by each path with the letters separating each panel representing the paths labelled in panel A. The horizontal white line indicates 0, and the horizontal black line indicates the overall mean effect size across all four leaf species.

The majority of direct effects of the three biological drivers on individual stream ecosystem processes were positive (shown as blue lines in Figure 3B–D and blue arrows in Figure 4) and consistent between most leaf litter species (shown as medium and thick arrows in Figure 4). Untransformed values of process rates are shown in Figure S7 and Table S1. Leaf litter in forested compared to non-forested sites tended to show higher nitrogen (N) loss (Figure 3C, c in Figure 4, Figure S1, Table S4, Table S6). There were on average (± SE) 12 (± 1.86), 18 (± 7.94), 22 (± 8.79), and 273 (± 60.95) % increases in N loss when comparing the 16 paired forested and non-forested sites for *Inga*, *Fraxinus*, *Alnus,* and *Miconia*, respectively (Figure S1, Table S7). Higher decomposition rates were associated with the presence of riparian forests across most leaf species (Figure 3B, Figure S1, Table S7); however, this pattern did not persist when accounting for all treatments and ecosystem processes in the SEM (b in Figure 4).

Macroinvertebrates overall had positive effects yet depending on the leaf species to differing extents (Figure 3B–D, Figure S1, Table S4, Table S6). Compared with the exclusion of macroinvertebrates, when both macroinvertebrate detritivores and decomposing microbes had access to the leaf litter bags, there was a clear increase in fungal biomass accrual on *Alnus* and *Fraxinus*, a slight increase on *Miconia*, and no effect on *Inga* (Figure 3D, d in Figure 4, Table S4, Table S6). Macroinvertebrates increased N loss for *Alnus*, *Fraxinus* and *Miconia*, but not *Inga* (Figure 3C, f in Figure 4, Table S4, Table S6).

Mixing different leaf species had mostly species-specific effects with both positive and negative effects for all three ecosystem processes (Figure 3C–D, g, h, i in Figure 4, Figure S1, Table S4, Table S6). In particular, *Miconia* showed higher fungal biomass accrual when together with *Inga*, while the opposite pattern occurred for *Inga* when together with *Miconia* (g in Figure 4, Figure 3D, Figure S1, Table S4, Table S6). For *Alnus* and *Fraxinus*, no such mixing effects occurred for fungal biomass accrual. Still, both leaf species had lower decomposition rates in mixed leaf litter bags (h in Figure 4, Figure 3B, Figure S1, Table S4, Table S6).

Ecosystem processes were positively associated amongst each other where credible intervals excluded zero. In other words, fungal biomass accrual, N loss, and decomposition rates were positively correlated in all leaf litter species, though the strength and certainty of these relationships varied (Figure S3, Table S5). However, several of these correlations had wide credible intervals in the SEM models, resulting in high uncertainty in the estimates and reflecting weak or limited relative importance compared to responses to the treatments (j, k, l in Figure 4).

## Discussion

Ecosystems are tightly connected across terrestrial and aquatic boundaries, and headwater streams exemplify this connectivity by responding strongly to changes in surrounding vegetation. The findings of this study show that processes in detrital food webs of headwater streams are enhanced by the presence of riparian forests, across both temperate and tropical regions. Forested stream sections consistently supported higher multifunctionality across all leaf litter species compared to non-forested sites. In addition, our findings highlight the central role of macroinvertebrate detritivores in supporting these ecosystem processes. Notably, both riparian forests and macroinvertebrates increased leaf litter nitrogen loss and decomposition rates. In contrast, the effects of leaf litter mixing were litter species dependent, suggesting that resource mixtures do not uniformly influence ecosystem processes. Nevertheless, the positive associations observed among ecosystem processes across regions indicate that key processes in detrital food webs may respond in similar and related ways, even under very different environmental conditions.

There were many similarities in how riparian forests, leaf litter and detritus consumers drive different aquatic-terrestrial ecosystem processes in headwaters (Figure 4). These similar patterns of ecosystem processes in detritus-based food webs across temperate and tropical headwater streams highlight the shared importance of cross-ecosystem linkages and common ecological drivers—particularly macroinvertebrates and riparian forests—in shaping these ecosystems and their functioning. Our insights agree with earlier studies with similar research questions but also expands our understanding of several ecosystem responses of two trophic levels in temperate and tropical stream reaches with and without riparian forests (Kominoski and others 2010; López-Rojo and others 2019). Given the scale and speed of current land-cover changes, further understanding of these cross-ecosystem linkages and drivers is essential not only for aquatic communities and their food web dynamics, but also for the many ecosystem processes they support. These findings also highlight the need for a holistic, cross-ecosystem perspective to fully understand, manage and conserve headwater streams.

Independent of biogeographic region, multifunctionality in detrital stream food webs was positively associated with riparian forests and macroinvertebrates, highlighting the important roles of both in sustaining these ecosystem dynamics (Figure 3A). In other words, the capacity of these headwater streams to support multiple ecosystem processes depended on the presence of riparian forests and macroinvertebrates (Cuffney and others 1990). These results suggest that the presence of higher trophic levels, such as macroinvertebrates, can strongly enhance ecosystem multifunctionality (Soliveres and others 2016) in headwater streams. Although temperate and tropical streams differ in many ecological characteristics (Boyero and others 2017, 2021; Shah et al. 2017), previous studies have found that microbial colonisation and growth on allochthonous leaf litter, detritivore feeding, and nutrient and decomposition dynamics may be shaped by similar abiotic and biotic drivers (Wantzen and Wagner 2006; Bruder and others 2014; Ferreira and others 2019). Importantly, many of these processes occur simultaneously and are partially interconnected (Figure S3; Table S5), meaning that changes in one process can propagate to others. This interdependence reinforces the concept of multifunctionality, where higher ecological complexity (for example, more trophic levels) promotes more complete resource use and thereby enhances overall ecosystem functioning (Kominoski and others 2010; Jabiol and others 2013; Oester and others 2023, 2024).

The predominantly positive effects of riparian forests on ecosystem processes in detrital food webs in both temperate and tropical streams highlight a key similarity between these systems. Besides the important effects of riparian forests on abiotic conditions of stream ecosystems, for example, regulation of shading, microclimate, sediment and nutrient retention, bank erosion, and hydrology (reviewed by Tolkkinen and others 2020; Ferreira and others 2023), riparian forests enhanced detritus-based nutrient cycling through increased N release from leaf litter. Possible explanations may lie in more favourable environmental conditions and resource availability (for example, detritus quantity and quality) for microbial decomposers and macroinvertebrate detritivores (Tolkkinen and others 2020). Indeed, the resources and habitats riparian forests provide for these functional groups are crucial (Wallace and others 1999; England and Rosemond 2004). Hence, the riparian conditions can directly or indirectly enhance decomposer and detritivore diversity, abundance, biomass, and activity, ultimately leading to increased leaf litter consumption rates and changes in stoichiometry of decomposing leaf litter (Casotti and others 2015; Oester and others 2023, 2024). Because N is only net released (mineralisation > immobilisation) in the later stages of leaf decay (García-Palacios and others 2017), a faster nutrient turnover indicates a higher N release from the resources to the environment. These released nutrients can be used by other consumers or reabsorbed by the riparian vegetation. However, it is worth noting that *Inga* leaves had not yet entered the mineralisation phase, as nitrogen content continued to increase throughout the experiment (that is, net-immobilisation during the experiment; Table S7). This likely reflects the early decomposition stage of *Inga* leaf litter at the end of the experiment (Table S3).

Similarly, also the presence of macroinvertebrates had predominantly positive effects on ecosystem processes in detrital food webs across both temperate and tropical streams, further reinforcing the functional parallels between these systems. While these results are in line with our hypotheses, as aquatic detritivores rely mostly on terrestrial resources (Cummins and others 1989; Danger and others 2012), these insights emphasise the great influence of macroinvertebrate detritivores on multiple ecosystem processes in both regions. This relatively strong effect aligns with studies showing that while environmental factors influence decomposition rates, macroinvertebrate detritivores often remain the primary agents of litter breakdown (Casotti and others 2015; Omoniyi and others 2021). While detritivore activity benefits from the presence and activity of microbes (Duarte and others 2006; Danger and others 2012), detritivores can also affect nutrient dynamics (and microbial activity) on the leaves, for example, through bioturbation and excretion (Wallace and Webster 1996; Chakraborty and others 2022). However, their importance for these ecosystem processes depends on the local biodiversity and abundances, especially in less species-rich regions, many of them in the neotropics (Wantzen and Wagner 2006; Bruder and others 2014; Handa and others 2014; Boyero and others 2021). Our results, showing strong effects of detritivores across sites, support the idea that local biodiversity can modulate these interactions and their contribution to ecosystem functioning.

The consequences of leaf litter mixing were species-specific and likely depended on the functional composition and traits of the leaf litter, influenced by differences in chemical composition (Schindler and Gessner 2009; Frainer and others 2015). Although all four litter species have been characterised as relatively fast decomposing species (Schindler and Gessner 2009; Bruder and others 2014; Kiffer and others 2018), they vary in chemical (Table S3) and physical characteristics, including toughness (Moretti and others 2007; Bruder and others 2014; Rezende and others 2019). *Inga* showed the lowest values across all ecosystem processes (Figure S1, Table S7). This may be attributed to *Inga*’s low nitrogen content and high toughness which requires greater energy investment from decomposers, thereby slowing decomposition rates and nutrient turnover (Moretti and others 2009). In Brazilian streams, selective consumption of higher-quality litter and the presence of recalcitrant compounds like lignin may have reduced overall processing. However, mixing *Inga* with *Miconia,* and *Alnus* with *Fraxinus* affected all three ecosystem processes. although untested here, These litter mixing effects might have also been mediated by macroinvertebrates as shown for other temperate (Santonja and others 2020) and tropical ecosystems (Rabelo and others 2024), as well as in global assessments (Handa and others 2014; Liu and others 2020). As riparian forests provide a variety of leaf litter (Wantzen and Wagner 2006; Little and Altermatt 2018), especially in the tropics (Boyero and others 2017; Rabelo and others 2024), it is important to assess the consequences of leaf litter mixing on multiple ecosystem processes to better understand the ecological complexity and sensitivity of headwater streams.

While we showed these food-web responses based on a two-level design of three relevant biological drivers on three major ecosystem processes in headwater streams (Dang and others 2005; Duarte and others 2006), considering continuous gradients of species diversity across multiple trophic levels (Soliveres and others 2016; López-Rojo and others 2019) and environmental conditions might result in even finer-scaled differentiation and “biodiversity fingerprints” in future studies (Lefcheck and othersand others 2015). As replicated field experiments across biomes are still uncommon (but see Wantzen and Wagner 2006; Bruder and others 2014; Handa and others 2014; Ferreira and others 2019; Tiegs and others 2019; Boyero and others 2021), and global meta-analyses on freshwater detrital food webs have only recently emerged (Ferreira and others 2016, 2019; Brauns et al. 2022; Oester and others 2025), our study contributes to cross-biome understanding of the drivers of ecosystem multifunctionality in detrital food webs of headwater streams.

Despite major environmental differences between temperate and tropical stream ecosystems, we found several mutual biological drivers of multiple ecosystem processes in their detrital food webs. Our study thus corroborates earlier ones in that multiple trophic levels as well as riparian forests are essential for stream ecosystems to fulfil their various roles. Thus, to understand the factors influencing ecosystem processes, it is essential to extend investigations beyond ecosystem boundaries when assessing the consequences of environmental change. Ultimately, a better understanding of the ecological role of small streams and their riparian vegetation as hotspots for biodiversity and ecosystem processes is key for their conservation in rapidly changing landscapes.

## Supplementary Information

Below is the link to the electronic supplementary material.

Supplementary file1 (DOCX 838 KB)

## Data Availability

The data and code that support the findings of this study are openly available on Dryad and Zenodo.

## Declarations

### Use of Artificial Intelligence (AI) and AI-assisted technologies

We have not used AI-assisted technologies in creating this article.
