# Supplementary material for: Riparian Forests and Macroinvertebrates Support Multiple Ecosystem Processes Across Temperate and Tropical Streams
Source: Ecosystems. 2025 Nov 10;29(1):3. doi: 10.1007/s10021-025-01024-0 (PMC12602634; doi:10.1007/s10021-025-01024-0)
Supplement: Supplementary file 1 — Supplementary file1 (DOCX 838 KB) [file 10021_2025_1024_MOESM1_ESM.docx]

**Supplementary Material**

**Riparian forests and macroinvertebrates support multiple ecosystem processes across temperate and tropical streams**

Rebecca Oester _1,2,3_,*, Paula M. de Omena _4_, Larissa Corteletti da Costa _4_, Marcelo S. Moretti _4_, Florian Altermatt _2,3_, Andreas Bruder _1_

**Affiliations:**1 Institute of Microbiology, University of Applied Sciences and Arts of Southern Switzerland, via Flora Ruchat Roncati 15, CH-6850 Mendrisio, Switzerland
2 Department of Evolutionary Biology and Environmental Studies, University of Zurich, Winterthurerstr. 190, CH-8057 Zurich, Switzerland
3 Eawag: Swiss Federal Institute of Aquatic Science and Technology, Department of Aquatic Ecology, Überlandstrasse 133, CH-8600 Dübendorf, Switzerland
4 Laboratory of Aquatic Insect Ecology, University of Vila Velha, Av. Comissário José Dantas de Melo 21, 29102-920 Vila Velha, ES, Brazil

* Corresponding author: [rebecca.oester@supsi.ch](mailto:rebecca.oester@supsi.ch)

**Table S1**: Hypotheses for the construction of the SEM meta-model. Each link between predictor and response variable is based on hypotheses derived from existing literature on temperate and/or tropical streams. The hypotheses represent either a positive (+), negative (-) or neutral (0) effect. Hypotheses for the drivers: riparian vegetation type (forested vs. non-forested), macroinvertebrate access (micro + macro vs. micro only), and leaf litter mixing (two leaf litter species in a mixture vs. single leaf litter species) are based on the difference between the reference state (non-forested, micro, single leaf species) and the more ecologically complex state (forested, micro+macro, mixed leaf species). For example, we expect higher decomposition rates in a forested site compared to a non-forested site, represented as a (+). Fungal biomass accrual is measured as net fungal biomass in the remaining leaf litter, N loss is measured as the % N released from the leaf litter, and decomposition rate is measured as k based on an exponential decay function described in the main text. The letters A-L correspond to Figure 4 in the main text.

| Variables | Link from | Ecosystem process | Letter | H_1_ CH | H_1_  BR | Sources |
| --- | --- | --- | --- | --- | --- | --- |
| exogeneous | Riparian vegetation type | Fungal biomass accrual | A | 0 | 0/+ | (1, 2) |
|  | Riparian vegetation type | Decomposition rate | B | + | + | (3–5) |
|  | Riparian vegetation type | Nitrogen loss | C | + | + | (6–9) |
|  | Macroinvertebrate access | Fungal biomass accrual | D | 0/- | 0 | (10–12) |
|  | Macroinvertebrate access | Decomposition rate | E | + | 0/+ | (13, 14) |
|  | Macroinvertebrate access | Nitrogen loss | F | + | 0 | (12, 15–18) |
|  | Leaf litter mixing | Fungal biomass accrual | G | +/- | +/- | (14, 19) |
|  | Leaf litter mixing | Decomposition rate | H | +/- | +/- | (13, 14, 20) |
|  | Leaf litter mixing | Nitrogen loss | I | +/- | +/- | (18, 20–22) |
| endogenous | Fungal biomass accrual | Nitrogen loss | J | 0 | - | (23–25) |
|  | Fungal biomass accrual | Decomposition rate | K | + | + | (23, 24) |
|  | Nitrogen loss | Decomposition rate | L | + | + | (24, 26) |

**Literature cited in Supplementary Material Table S1:**

1. C. Iñiguez‐Armijos, *et al.*, Shifts in leaf litter breakdown along a forest–pasture–urban gradient in Andean streams. *Ecology and Evolution* **6**, 4849–4865 (2016).

2. A. Lecerf, E. Chauvet, Diversity and functions of leaf-decaying fungi in human-altered streams. *Freshwater Biology* **53**, 1658–1672 (2008).

3. A. C. Encalada, J. Calles, V. Ferreira, C. M. Canhoto, M. a. S. Graça, Riparian land use and the relationship between the benthos and litter decomposition in tropical montane streams. *Freshwater Biology* **55**, 1719–1733 (2010).

4. J. S. Kominoski, L. B. Marczak, J. S. Richardson, Riparian forest composition affects stream litter decomposition despite similar microbial and invertebrate communities. *Ecology* **92**, 151–159 (2011).

5. R. Oester, P. C. dos Reis Oliveira, M. S. Moretti, F. Altermatt, A. Bruder, Leaf-associated macroinvertebrate assemblage and leaf litter breakdown in headwater streams depend on local riparian vegetation. *Hydrobiologia* **850**, 3359–3374 (2023).

6. J. R. Webster, J. D. Newbold, S. A. Thomas, H. M. Valett, P. J. Mulholland, Nutrient Uptake and Mineralization during Leaf Decay in Streams – a Model Simulation. *International Review of Hydrobiology* **94**, 372–390 (2009).

7. A. Martínez, A. Larrañaga, J. Pérez, A. Basaguren, J. Pozo, Leaf-litter quality effects on stream ecosystem functioning: A comparison among five species. *Fundamental and Applied Limnology / Archiv für Hydrobiologie* **183**, 239–248 (2013).

8. J. Rubio-Ríos, *et al.*, Alder stands promote N-cycling but not leaf litter mass loss in Mediterranean streams flowing through pine plantations. *Forest Ecology and Management* **542**, 121072 (2023).

9. S. Li, *et al.*, Litter decomposition and nutrient release are faster under secondary forests than under Chinese fir plantations with forest development. *Sci Rep* **13**, 16805 (2023).

10. T. L. Arsuffi, K. Suberkropp, Selective feeding by shredders on leaf-colonizing stream fungi: comparison of macroinvertebrate taxa. *Oecologia* **79**, 30–37 (1989).

11. J. Jabiol, *et al.*, Diversity patterns of leaf-associated aquatic hyphomycetes along a broad latitudinal gradient. *Fungal Ecology* **6**, 439–448 (2013).

12. V. D. Villanueva, R. Albariño, C. Canhoto, Positive effect of shredders on microbial biomass and decomposition in stream microcosms. *Freshwater Biology* **57**, 2504–2513 (2012).

13. A. Bruder, M. H. Schindler, M. S. Moretti, M. O. Gessner, Litter decomposition in a temperate and a tropical stream: the effects of species mixing, litter quality and shredders. *Freshwater Biology* **59**, 438–449 (2014).

14. V. Ferreira, A. C. Encalada, M. A. S. Graça, Effects of litter diversity on decomposition and biological colonization of submerged litter in temperate and tropical streams. *Freshwater Science* **31**, 945–962 (2012).

15. J. P. Benstead, J. G. March, C. M. Pringle, K. C. Ewel, J. W. Short, Biodiversity and ecosystem function in species-poor communities: community structure and leaf litter breakdown in a Pacific island stream. *Journal of the North American Benthological Society* **28**, 454–465 (2009).

16. M. A. McCary, O. J. Schmitz, Invertebrate functional traits and terrestrial nutrient cycling: insights from a global meta-analysis. *Journal of Animal Ecology* **n/a** (2021).

17. M. J. Vanni, Nutrient Cycling by Animals in Freshwater Ecosystems. *Annu. Rev. Ecol. Syst.* **33**, 341–370 (2002).

18. I. T. Handa, *et al.*, Consequences of biodiversity loss for litter decomposition across biomes. *Nature* **509**, 218–221 (2014).

19. S. Duarte, C. Pascoal, F. Cássio, F. Bärlocher, Aquatic hyphomycete diversity and identity affect leaf litter decomposition in microcosms. *Oecologia* **147**, 658–666 (2006).

20. T. B. Gartner, Z. G. Cardon, Decomposition dynamics in mixed-species leaf litter. *Oikos* **104**, 230–246 (2004).

21. J. S. Kominoski, T. J. Hoellein, J. J. Kelly, C. M. Pringle, Does mixing litter of different qualities alter stream microbial diversity and functioning on individual litter species? *Oikos* **118**, 457–463 (2009).

22. N. E. Pettit, *et al.*, Leaf litter chemistry, decomposition and assimilation by macroinvertebrates in two tropical streams. *Hydrobiologia* **680**, 63–77 (2012).

23. V. Gulis, R. Su, K. A. Kuehn, “Fungal Decomposers in Freshwater Environments” in *The Structure and Function of Aquatic Microbial Communities*, Advances in Environmental Microbiology., C. J. Hurst, Ed. (Springer International Publishing, 2019), pp. 121–155.

24. S. Hladyz, M. O. Gessner, P. S. Giller, J. Pozo, G. Woodward, Resource quality and stoichiometric constraints on stream ecosystem functioning. *Freshwater Biology* **54**, 957–970 (2009).

25. P. García-Palacios, B. G. McKie, I. T. Handa, A. Frainer, S. Hättenschwiler, The importance of litter traits and decomposers for litter decomposition: a comparison of aquatic and terrestrial ecosystems within and across biomes. *Functional Ecology* **30**, 819–829 (2016).

26. C. J. Robbins, *et al.*, Nutrient and stoichiometry dynamics of decomposing litter in stream ecosystems: A global synthesis. *Ecology* **104**, e4060 (2023).

**Table S2**: Site characteristic from measurements at the start and end of field experiment averaged across the 16 sites in Switzerland and Brazil. The numbers represent the mean ± standard deviation. b.d. indicates “below detection limit”, which was 0.07 µg L^-1^ for PO_4_^3-^ and 2.00 mgL^-1^ for DOC.

| Variable | Switzerland | | Brazil | |
| --- | --- | --- | --- | --- |
|  | forested | non-forested | forested | non-forested |
| Temperature [°C] | 4.21±1.15 | 4.41±1.40 | 20.86±1.75 | 21.15±1.84 |
| Velocity [ms^-1^] | 0.19±0.05 | 0.24±0.11 | 0.19±0.06 | 0.19±0.06 |
| pH | 7.58±0.51 | 7.56±0.46 | 6.76±0.11 | 6.68±0.32 |
| Conductivity [µScm^-1^] | 190.00±143.51 | 196.75±143.54 | 49.00±10.46 | 51.06±10.12 |
| NO_3_^-^ [mgL^-1^**]** | 2.44±2.19 | 2.22±2.20 | 0.76±0.29 | 0.79±0.32 |
| PO_4_^3-^ [µgL^-1^] | 7.87±1.95 | 3.82±8.62 | b.d. | b.d. |
| DOC [mgL^-1^] | 2.65±2.07 | 2.74±2.15 | b.d. | b.d. |
| O_2_ [%] | 98.94±1.41 | 99.29±2.54 | 106.93±11.61 | 102.41±8.87 |
| Alkalinity [mmolL^-1^] | 1.80±1.44 | 1.93±1.43 | 0.06±0.02 | 0.05±0.01 |
| Hardness [mmolL^-1^] | 1.16±0.83 | 1.22±0.82 | 2.44±1.08 | 2.64±0.81 |

**Table S3**: Initial leaf litter characteristics. Mean ± standard deviation.

| Taxon | %N | %C | %P | % fibre | % lignin |
| --- | --- | --- | --- | --- | --- |
| *Alnus* | 2.15±0.13 | 48.58±0.78 | 0.07±0.007 | 39.73±3.37 | 25.83±4.19 |
| *Fraxinus* | 1.52±0.23 | 42.71±4.64 | 0.09±0.007 | 36.59±3.52 | 19.57±5.04 |
| *Inga* | 0.90±0.28 | 33.32±8.38 | 0.02±0.001 | 76.31±0.91 | 49.39±0.75 |
| *Miconia* | 1.24±0.21 | 37.45±5.53 | 0.01±0.001 | 40.87±0.35 | 24.78±0.47 |

**Table S4**: Detailed model outputs for treatment effects on each process separately and as multifunctionality.

| Coefficient | Estimate | EstError | l-95 | u-95 | Rhat | Bulk_ESS | Tail_ESS | Taxon | Model |
| --- | --- | --- | --- | --- | --- | --- | --- | --- | --- |
| Intercept | -0.18 | 0.25 | -0.77 | 0.34 | 1.00 | 1435 | 1511 | *Alnus* | meanFunction ~ Mesh + Vegetation + Mix |
| micro+macro | **0.43** | 0.04 | **0.35** | **0.51** | 1.00 | 4137 | 2589 | *Alnus* | meanFunction ~ Mesh + Vegetation + Mix |
| forested | **0.13** | 0.04 | **0.05** | **0.21** | 1.00 | 4155 | 2908 | *Alnus* | meanFunction ~ Mesh + Vegetation + Mix |
| mixed | -0.07 | 0.04 | -0.15 | 0.01 | 1.00 | 4318 | 3080 | *Alnus* | meanFunction ~ Mesh + Vegetation + Mix |
| Intercept | 0.11 | 0.45 | -0.82 | 0.81 | 1.00 | 2912 | 2128 | *Alnus* | biomass ~ Mesh + Vegetation + Mix |
| micro+macro | **0.44** | 0.05 | **0.35** | **0.53** | 1.00 | 5178 | 2455 | *Alnus* | biomass ~ Mesh + Vegetation + Mix |
| forested | **-0.11** | 0.05 | **-0.20** | **-0.02** | 1.00 | 4900 | 2801 | *Alnus* | biomass ~ Mesh + Vegetation + Mix |
| mixed | -0.05 | 0.05 | -0.14 | 0.04 | 1.00 | 4747 | 2877 | *Alnus* | biomass ~ Mesh + Vegetation + Mix |
| Intercept | -0.73 | 0.40 | -1.29 | 0.21 | 1.00 | 1019 | 1883 | *Alnus* | N loss ~ Mesh + Vegetation + Mix |
| micro+macro | **0.56** | 0.08 | **0.39** | **0.72** | 1.00 | 4284 | 2851 | *Alnus* | N loss ~ Mesh + Vegetation + Mix |
| forested | **0.23** | 0.08 | **0.06** | **0.39** | 1.00 | 4151 | 2918 | *Alnus* | N loss ~ Mesh + Vegetation + Mix |
| mixed | -0.14 | 0.08 | -0.30 | 0.01 | 1.00 | 4251 | 2796 | *Alnus* | N loss ~ Mesh + Vegetation + Mix |
| Intercept | -0.50 | 0.33 | -1.11 | 0.26 | 1.00 | 1758 | 1977 | *Alnus* | k ~ Mesh + Vegetation + Mix |
| micro+macro | 0.61 | 0.07 | **0.47** | **0.75** | 1.00 | 4011 | 2649 | *Alnus* | k ~ Mesh + Vegetation + Mix |
| forested | 0.30 | 0.07 | **0.16** | **0.44** | 1.00 | 4515 | 2838 | *Alnus* | k ~ Mesh + Vegetation + Mix |
| mixed | **-0.25** | 0.07 | **-0.39** | **-0.11** | 1.00 | 4566 | 2428 | *Alnus* | k ~ Mesh + Vegetation + Mix |
| Intercept | -0.38 | 0.25 | -0.93 | 0.18 | 1.00 | 1298 | 1108 | *Fraxinus* | meanFunction ~ Mesh + Vegetation + Mix |
| micro+macro | **0.48** | 0.04 | **0.39** | **0.56** | 1.00 | 3922 | 2627 | *Fraxinus* | meanFunction ~ Mesh + Vegetation + Mix |
| forested | **0.13** | 0.04 | **0.05** | **0.21** | 1.00 | 4208 | 2810 | *Fraxinus* | meanFunction ~ Mesh + Vegetation + Mix |
| mixed | -0.00 | 0.04 | -0.08 | 0.08 | 1.00 | 4161 | 2698 | *Fraxinus* | meanFunction ~ Mesh + Vegetation + Mix |
| Intercept | 0.07 | 0.45 | -0.87 | 0.80 | 1.00 | 4045 | 2088 | *Fraxinus* | biomass ~ Mesh + Vegetation + Mix |
| micro+macro | **0.34** | 0.06 | **0.23** | **0.45** | 1.00 | 5113 | 2842 | *Fraxinus* | biomass ~ Mesh + Vegetation + Mix |
| forested | -0.10 | 0.06 | -0.22 | 0.01 | 1.00 | 4809 | 3034 | *Fraxinus* | biomass ~ Mesh + Vegetation + Mix |
| mixed | 0.03 | 0.06 | -0.08 | 0.14 | 1.00 | 4570 | 2872 | *Fraxinus* | biomass ~ Mesh + Vegetation + Mix |
| Intercept | -1.09 | 0.42 | -1.65 | -0.11 | 1.00 | 926 | 1803 | *Fraxinus* | N loss ~ Mesh + Vegetation + Mix |
| micro+macro | **0.84** | 0.08 | **0.67** | **0.98** | 1.00 | 2645 | 1524 | *Fraxinus* | N loss ~ Mesh + Vegetation + Mix |
| forested | **0.35** | 0.09 | **0.17** | **0.52** | 1.00 | 3645 | 2618 | *Fraxinus* | N loss ~ Mesh + Vegetation + Mix |
| mixed | 0.05 | 0.09 | -0.13 | 0.22 | 1.00 | 3875 | 2438 | *Fraxinus* | N loss ~ Mesh + Vegetation + Mix |
| Intercept | -0.92 | 0.42 | -1.54 | 0.03 | 1.00 | 1489 | 2070 | *Fraxinus* | k ~ Mesh + Vegetation + Mix |
| micro+macro | **0.80** | 0.08 | **0.64** | **0.94** | 1.00 | 2605 | 1506 | *Fraxinus* | k ~ Mesh + Vegetation + Mix |
| forested | **0.31** | 0.08 | **0.15** | **0.47** | 1.00 | 3767 | 2425 | *Fraxinus* | k ~ Mesh + Vegetation + Mix |
| mixed | -0.03 | 0.08 | -0.18 | 0.13 | 1.00 | 3792 | 2583 | *Fraxinus* | k ~ Mesh + Vegetation + Mix |
| Intercept | -0.13 | 0.24 | -0.63 | 0.45 | 1.00 | 1805 | 1486 | *Inga* | meanFunction ~ Mesh + Vegetation + Mix |
| micro+macro | 0.00 | 0.03 | -0.06 | 0.07 | 1.00 | 4140 | 2748 | *Inga* | meanFunction ~ Mesh + Vegetation + Mix |
| forested | **0.08** | 0.03 | **0.01** | **0.14** | 1.00 | 4643 | 3055 | *Inga* | meanFunction ~ Mesh + Vegetation + Mix |
| mixed | 0.04 | 0.03 | -0.03 | 0.11 | 1.00 | 4207 | 2595 | *Inga* | meanFunction ~ Mesh + Vegetation + Mix |
| Intercept | 0.36 | 0.43 | -0.56 | 1.05 | 1.00 | 2600 | 2517 | *Inga* | biomass ~ Mesh + Vegetation + Mix |
| micro+macro | -0.01 | 0.07 | -0.15 | 0.14 | 1.00 | 4469 | 2634 | *Inga* | biomass ~ Mesh + Vegetation + Mix |
| forested | 0.11 | 0.07 | -0.04 | 0.26 | 1.00 | 4181 | 2713 | *Inga* | biomass ~ Mesh + Vegetation + Mix |
| mixed | **-0.25** | 0.07 | **-0.40** | **-0.11** | 1.00 | 4754 | 2506 | *Inga* | biomass ~ Mesh + Vegetation + Mix |
| Intercept | -0.60 | 0.42 | -1.14 | 0.39 | 1.01 | 1047 | 1784 | *Inga* | N loss ~ Mesh + Vegetation + Mix |
| micro+macro | -0.01 | 0.09 | -0.17 | 0.16 | 1.00 | 4179 | 2841 | *Inga* | N loss ~ Mesh + Vegetation + Mix |
| forested | 0.07 | 0.08 | -0.09 | 0.24 | 1.00 | 4468 | 2742 | *Inga* | N loss ~ Mesh + Vegetation + Mix |
| mixed | **0.23** | 0.08 | **0.07** | **0.39** | 1.00 | 4680 | 2934 | *Inga* | N loss ~ Mesh + Vegetation + Mix |
| Intercept | -0.50 | 0.37 | -1.06 | 0.39 | 1.00 | 1145 | 1650 | *Inga* | k ~ Mesh + Vegetation + Mix |
| micro+macro | 0.11 | 0.06 | -0.00 | 0.23 | 1.01 | 4283 | 2835 | *Inga* | k ~ Mesh + Vegetation + Mix |
| forested | 0.11 | 0.06 | -0.01 | 0.24 | 1.00 | 4829 | 2548 | *Inga* | k ~ Mesh + Vegetation + Mix |
| mixed | 0.09 | 0.06 | -0.03 | 0.21 | 1.00 | 4317 | 2826 | *Inga* | k ~ Mesh + Vegetation + Mix |
| Intercept | -0.24 | 0.29 | -0.93 | 0.28 | 1.00 | 1842 | 2088 | *Miconia* | meanFunction ~ Mesh + Vegetation + Mix |
| micro+macro | **0.52** | 0.05 | **0.41** | **0.62** | 1.00 | 4793 | 2621 | *Miconia* | meanFunction ~ Mesh + Vegetation + Mix |
| forested | **0.19** | 0.05 | **0.09** | **0.29** | 1.00 | 4308 | 2595 | *Miconia* | meanFunction ~ Mesh + Vegetation + Mix |
| mixed | 0.08 | 0.05 | -0.02 | 0.19 | 1.00 | 4312 | 2818 | *Miconia* | meanFunction ~ Mesh + Vegetation + Mix |
| Intercept | -0.08 | 0.43 | -1.00 | 0.59 | 1.00 | 1645 | 1686 | *Miconia* | biomass ~ Mesh + Vegetation + Mix |
| micro+macro | **0.15** | 0.06 | **0.03** | **0.27** | 1.00 | 4393 | 2454 | *Miconia* | biomass ~ Mesh + Vegetation + Mix |
| forested | 0.08 | 0.06 | -0.04 | 0.20 | 1.00 | 4656 | 2820 | *Miconia* | biomass ~ Mesh + Vegetation + Mix |
| mixed | **0.53** | 0.06 | **0.41** | **0.65** | 1.00 | 4148 | 2240 | *Miconia* | biomass ~ Mesh + Vegetation + Mix |
| Intercept | -0.69 | 0.30 | -1.26 | 0.02 | 1.00 | 1586 | 1671 | *Miconia* | N loss ~ Mesh + Vegetation + Mix |
| micro+macro | **0.80** | 0.09 | **0.61** | **0.97** | 1.00 | 2607 | 1266 | *Miconia* | N loss ~ Mesh + Vegetation + Mix |
| forested | **0.39** | 0.10 | **0.20** | **0.58** | 1.00 | 3419 | 2557 | *Miconia* | N loss ~ Mesh + Vegetation + Mix |
| mixed | -0.05 | 0.10 | -0.23 | 0.14 | 1.00 | 4076 | 2517 | *Miconia* | N loss ~ Mesh + Vegetation + Mix |
| Intercept | -0.52 | 0.33 | -1.13 | 0.21 | 1.00 | 2414 | 2214 | *Miconia* | k ~ Mesh + Vegetation + Mix |
| micro+macro | **0.62** | 0.08 | **0.47** | **0.76** | 1.00 | 4281 | 2306 | *Miconia* | k ~ Mesh + Vegetation + Mix |
| forested | **0.28** | 0.08 | **0.13** | **0.44** | 1.01 | 4229 | 2080 | *Miconia* | k ~ Mesh + Vegetation + Mix |
| mixed | -0.07 | 0.08 | -0.22 | 0.08 | 1.00 | 4516 | 2701 | *Miconia* | k ~ Mesh + Vegetation + Mix |

**Table S5**: Detailed model outputs for each ecosystem function among themselves. Corresponding correlation plots are shown in Figure S3.

| Coeffient | Estimate | EstError | l-95 | u-95 | Rhat | Bulk_ESS |  | Tail_ESS | Taxon | Model |
| --- | --- | --- | --- | --- | --- | --- | --- | --- | --- | --- |
| Intercept | 0.15 | 0.43 | -0.74 | 0.86 | 1.00 | 2343 |  | 1606 | *Alnus* | Biomass ~ N loss |
| N_Loss.std | 0.33 | 0.20 | -0.07 | 0.72 | 1.00 | 3824 |  | 1889 | *Alnus* | Biomass ~ N loss |
| Intercept | 0.15 | 0.44 | -0.74 | 0.89 | 1.00 | 2998 |  | 1846 | *Fraxinus* | Biomass ~ N loss |
| N_Loss.std | 0.18 | 0.20 | -0.20 | 0.56 | 1.00 | 3859 |  | 2625 | *Fraxinus* | Biomass ~ N loss |
| Intercept | 0.35 | 0.45 | -0.62 | 1.04 | 1.00 | 1965 |  | 1993 | *Inga* | Biomass ~ N loss |
| N_Loss.std | -0.23 | 0.27 | -0.76 | 0.32 | 1.00 | 3065 |  | 1818 | *Inga* | Biomass ~ N loss |
| Intercept | 0.08 | 0.43 | -0.86 | 0.77 | 1.00 | 1577 |  | 1692 | *Miconia* | Biomass ~ N loss |
| N_Loss.std | **0.51** | 0.18 | **0.17** | **0.86** | 1.00 | 2685 |  | 1399 | *Miconia* | Biomass ~ N loss |
| Intercept | -0.10 | 0.44 | -1.03 | 0.60 | 1.00 | 2415 |  | 2172 | *Alnus* | Biomass ~ k |
| logk.std | **0.82** | 0.13 | **0.50** | **0.99** | 1.00 | 2771 |  | 1788 | *Alnus* | Biomass ~ k |
| Intercept | 0.08 | 0.43 | -0.84 | 0.81 | 1.00 | 2877 |  | 2075 | *Fraxinus* | Biomass ~ k |
| logk.std | 0.41 | 0.23 | -0.03 | 0.85 | 1.00 | 2994 |  | 1855 | *Fraxinus* | Biomass ~ k |
| Intercept | 0.08 | 0.44 | -0.85 | 0.81 | 1.00 | 1742 |  | 1671 | *Inga* | Biomass ~ k |
| logk.std | 0.56 | 0.28 | -0.06 | 0.98 | 1.00 | 2818 |  | 1287 | *Inga* | Biomass ~ k |
| Intercept | -0.05 | 0.44 | -1.02 | 0.61 | 1.00 | 1406 |  | 1903 | *Miconia* | Biomass ~ k |
| logk.std | **0.85** | 0.12 | **0.54** | **0.99** | 1.00 | 2379 |  | 1464 | *Miconia* | Biomass ~ k |
| Intercept | -0.47 | 0.31 | -1.05 | 0.25 | 1.00 | 2045 |  | 2077 | *Alnus* | k ~ N loss |
| N_Loss.std | **0.98** | 0.02 | **0.92** | **1.00** | 1.00 | 2593 |  | 1605 | *Alnus* | k ~ N loss |
| Intercept | -0.65 | 0.42 | -1.23 | 0.32 | 1.00 | 1224 |  | 1856 | *Fraxinus* | k ~ N loss |
| N_Loss.std | **0.98** | 0.02 | **0.92** | **1.00** | 1.00 | 2050 |  | 1268 | *Fraxinus* | k ~ N loss |
| Intercept | -0.62 | 0.35 | -1.14 | 0.23 | 1.00 | 1394 |  | 2078 | *Inga* | k ~ N loss |
| N_Loss.std | **0.95** | 0.04 | **0.84** | **1.00** | 1.00 | 2618 |  | 1537 | *Inga* | k ~ N loss |
| Intercept | -0.56 | 0.31 | -1.15 | 0.13 | 1.00 | 1564 |  | 1513 | *Miconia* | k ~ N loss |
| N_Loss.std | **0.98** | 0.02 | **0.93** | **1.00** | 1.00 | 2908 |  | 1483 | *Miconia* | k ~ N loss |

**Table S6**: Detailed model outputs for SEMs.

| Coefficient | Estimate | Est.Error | l95 | u05 | Rhat | Bulk_ESS | Tail_ESS | Taxon | Letter |
| --- | --- | --- | --- | --- | --- | --- | --- | --- | --- |
| Intercept k | -0.13 | 0.55 | -1.28 | 1.02 | 1.00 | 11728 | 11295 | *Alnus* | Intercept |
| Intercept Nloss | -0.43 | 0.51 | -1.52 | 0.68 | 1.00 | 12304 | 11648 | *Alnus* | Intercept |
| Intercept biomass | -0.28 | 0.58 | -1.46 | 0.92 | 1.00 | 13036 | 13025 | *Alnus* | Intercept |
| k(micro+macro) | 0.34 | 0.24 | -0.13 | 0.82 | 1.00 | 8364 | 10368 | *Alnus* | E |
| k(mix) | **-0.23** | 0.07 | **-0.37** | **-0.10** | 1.00 | 16222 | 13189 | *Alnus* | H |
| k(forested) | 0.16 | 0.12 | -0.07 | 0.41 | 1.00 | 6318 | 8846 | *Alnus* | B |
| k(biomass) | 0.25 | 0.24 | -0.22 | 0.73 | 1.00 | 6286 | 9017 | *Alnus* | K |
| k(Nloss) | 0.50 | 0.27 | -0.05 | 1.03 | 1.00 | 5600 | 7756 | *Alnus* | L |
| Nloss(micro+macro) | **0.57** | 0.15 | **0.27** | **0.86** | 1.00 | 13499 | 13752 | *Alnus* | F |
| Nloss(mix) | -0.07 | 0.11 | -0.29 | 0.16 | 1.00 | 23490 | 14256 | *Alnus* | I |
| Nloss(forested) | **0.36** | 0.12 | **0.13** | **0.59** | 1.00 | 24614 | 14992 | *Alnus* | C |
| Nloss(biomass) | 0.26 | 0.14 | -0.03 | 0.53 | 1.00 | 10706 | 10279 | *Alnus* | J |
| biomass(micro+macro) | **0.73** | 0.08 | **0.59** | **0.88** | 1.00 | 27887 | 15759 | *Alnus* | D |
| biomass(mix) | -0.06 | 0.08 | -0.20 | 0.09 | 1.00 | 23189 | 15281 | *Alnus* | G |
| biomass(forested) | -0.14 | 0.07 | -0.29 | 0.00 | 1.00 | 26258 | 15529 | *Alnus* | A |
| Intercept k | -0.08 | 0.52 | -1.18 | 1.01 | 1.00 | 13213 | 12821 | *Fraxinus* | Intercept |
| Intercept Nloss | -0.67 | 0.48 | -1.74 | 0.40 | 1.00 | 14286 | 11712 | *Fraxinus* | Intercept |
| Intercept biomass | -0.26 | 0.59 | -1.52 | 0.98 | 1.00 | 16003 | 13545 | *Fraxinus* | Intercept |
| k(micro+macro | 0.19 | 0.28 | -0.35 | 0.78 | 1.00 | 9189 | 11370 | *Fraxinus* | E |
| k(mix) | **-0.16** | 0.07 | **-0.29** | **-0.03** | 1.00 | 17803 | 13019 | *Fraxinus* | H |
| k(forested) | 0.13 | 0.11 | -0.07 | 0.38 | 1.00 | 7610 | 10104 | *Fraxinus* | B |
| k(biomass) | 0.21 | 0.25 | -0.27 | 0.72 | 1.00 | 6834 | 10685 | *Fraxinus* | K |
| k(Nloss) | **0.61** | 0.26 | **0.06** | **1.08** | 1.00 | 7211 | 9576 | *Fraxinus* | L |
| Nloss(micro+macro) | 0.96 | 0.16 | **0.62** | **1.26** | 1.00 | 11875 | 12414 | *Fraxinus* | F |
| Nloss(mix) | 0.06 | 0.11 | -0.15 | 0.27 | 1.00 | 23933 | 15307 | *Fraxinus* | I |
| Nloss(forested) | **0.33** | 0.11 | **0.11** | **0.56** | 1.00 | 22584 | 15511 | *Fraxinus* | C |
| Nloss(biomass) | 0.12 | 0.21 | -0.27 | 0.56 | 1.00 | 9166 | 9776 | *Fraxinus* | J |
| biomass(micro+macro) | **0.60** | 0.10 | **0.41** | **0.79** | 1.00 | 28958 | 15214 | *Fraxinus* | D |
| biomass(mix) | 0.07 | 0.10 | -0.12 | 0.26 | 1.00 | 25437 | 15564 | *Fraxinus* | G |
| biomass(forested) | -0.17 | 0.09 | -0.35 | 0.02 | 1.00 | 29177 | 15399 | *Fraxinus* | A |
| Intercept k | -0.22 | 0.53 | -1.32 | 0.91 | 1.00 | 11042 | 12024 | *Inga* | Intercept |
| Intercept Nloss | -0.25 | 0.45 | -1.25 | 0.72 | 1.00 | 11841 | 11205 | *Inga* | Intercept |
| Intercept biomass | 0.18 | 0.53 | -0.95 | 1.28 | 1.00 | 11386 | 12178 | *Inga* | Intercept |
| k(micro+macro) | 0.08 | 0.10 | -0.12 | 0.28 | 1.00 | 20795 | 14798 | *Inga* | E |
| k(mix) | 0.20 | 0.20 | -0.20 | 0.60 | 1.00 | 6992 | 11405 | *Inga* | H |
| k(forested) | 0.15 | 0.12 | -0.08 | 0.39 | 1.00 | 12007 | 12999 | *Inga* | B |
| k(biomass) | 0.33 | 0.32 | -0.32 | 0.95 | 1.00 | 6151 | 9769 | *Inga* | K |
| k(Nloss) | 0.23 | 0.33 | -0.46 | 0.89 | 1.00 | 6258 | 9339 | *Inga* | L |
| Nloss(micro+macro) | -0.08 | 0.12 | -0.31 | 0.15 | 1.00 | 30820 | 16454 | *Inga* | F |
| Nloss(mix) | **0.38** | 0.15 | **0.09** | **0.68** | 1.00 | 14319 | 14014 | *Inga* | I |
| Nloss(forested) | 0.19 | 0.12 | -0.04 | 0.42 | 1.00 | 28382 | 15065 | *Inga* | C |
| Nloss(biomass) | -0.03 | 0.23 | -0.50 | 0.41 | 1.00 | 8043 | 9482 | *Inga* | J |
| biomass(micro+macro) | -0.01 | 0.11 | -0.22 | 0.20 | 1.00 | 30341 | 16554 | *Inga* | D |
| biomass(mix) | **-0.42** | 0.11 | **-0.63** | **-0.21** | 1.00 | 34384 | 15007 | *Inga* | G |
| biomass(forested) | 0.06 | 0.11 | -0.15 | 0.27 | 1.00 | 30942 | 15076 | *Inga* | A |
| Intercept k | 0.12 | 0.50 | -0.97 | 1.19 | 1.00 | 11313 | 11347 | *Miconia* | Intercept |
| Intercept Nloss | -0.44 | 0.51 | -1.52 | 0.66 | 1.00 | 12554 | 11039 | *Miconia* | Intercept |
| Intercept biomass | -0.57 | 0.53 | -1.68 | 0.54 | 1.00 | 13124 | 12419 | *Miconia* | Intercept |
| k(micro+macro) | 0.15 | 0.22 | -0.27 | 0.59 | 1.00 | 7305 | 8825 | *Miconia* | E |
| k(mix) | -0.36 | 0.19 | -0.73 | 0.03 | 1.00 | 6475 | 9890 | *Miconia* | H |
| k(forested) | -0.04 | 0.10 | -0.24 | 0.17 | 1.00 | 9320 | 11466 | *Miconia* | B |
| k(biomass) | 0.40 | 0.23 | -0.06 | 0.83 | 1.00 | 5912 | 9280 | *Miconia* | K |
| k(Nloss) | **0.67** | 0.23 | **0.21** | **1.10** | 1.00 | 7082 | 8928 | *Miconia* | L |
| Nloss(micro+macro) | **0.91** | 0.11 | **0.69** | **1.11** | 1.00 | 20482 | 13830 | *Miconia* | F |
| Nloss(mix) | -0.33 | 0.21 | -0.73 | 0.10 | 1.00 | 7632 | 10907 | *Miconia* | I |
| Nloss(forested) | **0.30** | 0.11 | **0.08** | **0.51** | 1.00 | 17570 | 14539 | *Miconia* | C |
| Nloss(biomass) | 0.31 | 0.24 | -0.20 | 0.76 | 1.00 | 6687 | 9566 | *Miconia* | J |
| biomass(micro+macro) | 0.15 | 0.09 | -0.03 | 0.34 | 1.00 | 30745 | 14508 | *Miconia* | D |
| biomass(mix) | **0.80** | 0.09 | **0.62** | **0.99** | 1.00 | 30503 | 14538 | *Miconia* | G |
| biomass(forested) | **0.19** | 0.09 | **0.01** | **0.37** | 1.00 | 29440 | 15710 | *Miconia* | A |

**Table S7**: Untransformed mean ± SD for each ecosystem function, treatment and across all four leaf species *Alnus*, *Fraxinus*, *Inga* and *Miconia*.

| Function | Treatment | *Alnus* | | *Fraxinus* | | *Inga* | | *Miconia* | |
| --- | --- | --- | --- | --- | --- | --- | --- | --- | --- |
|  |  | Mean | SD | Mean | SD | Mean | SD | Mean | SD |
| Decomposition rates [dd-1] | mono non-forested micro | 0.00386 | 0.00079 | 0.00649 | 0.00116 | 0.00019 | 0.00004 | 0.00047 | 0.00013 |
|  | mix non-forested micro | 0.00343 | 0.00079 | 0.00631 | 0.00148 | 0.00019 | 0.00004 | 0.00052 | 0.00018 |
|  | mono non-forested micro+macro | 0.00446 | 0.00078 | 0.00762 | 0.00127 | 0.00019 | 0.00004 | 0.00086 | 0.00063 |
|  | mix non-forested micro+macro | 0.00415 | 0.00073 | 0.00767 | 0.00152 | 0.00019 | 0.00004 | 0.00072 | 0.00054 |
|  | mono forested micro | 0.00364 | 0.00061 | 0.00637 | 0.00083 | 0.00019 | 0.00003 | 0.00051 | 0.00015 |
|  | mix forested micro | 0.00348 | 0.00059 | 0.00624 | 0.00099 | 0.00021 | 0.00006 | 0.00048 | 0.00016 |
|  | mono forested micro+macro | 0.00534 | 0.00159 | 0.00956 | 0.00350 | 0.00021 | 0.00009 | 0.00116 | 0.00079 |
|  | mix forested micro+macro | 0.00513 | 0.00205 | 0.00916 | 0.00381 | 0.00023 | 0.00017 | 0.00101 | 0.00063 |
| Fungal biomass accrual [mg/g] | mono non-forested micro | 60.88 | 25.20 | 80.11 | 22.88 | 22.74 | 6.86 | 18.13 | 8.68 |
|  | mix non-forested micro | 62.27 | 26.22 | 83.81 | 19.12 | 18.31 | 5.90 | 29.50 | 8.73 |
|  | mono non-forested micro+macro | 92.60 | 36.40 | 106.87 | 28.38 | 22.59 | 7.16 | 21.96 | 10.96 |
|  | mix non-forested micro+macro | 86.29 | 22.22 | 101.56 | 20.75 | 19.12 | 6.32 | 30.40 | 11.40 |
|  | mono forested micro | 63.01 | 24.10 | 80.64 | 20.80 | 22.02 | 6.83 | 22.36 | 8.71 |
|  | mix forested micro | 60.73 | 19.22 | 85.85 | 23.81 | 21.15 | 8.92 | 28.63 | 8.23 |
|  | mono forested micro+macro | 76.40 | 21.97 | 94.02 | 25.71 | 22.45 | 6.56 | 23.48 | 9.52 |
|  | mix forested micro+macro | 75.77 | 27.52 | 94.22 | 28.70 | 19.04 | 5.85 | 30.30 | 9.97 |
| N loss [%] | mono non-forested micro | 10.94 | 5.01 | 16.08 | 8.52 | -21.06 | 10.02 | -2.04 | 20.79 |
|  | mix non-forested micro | 10.32 | 7.12 | 14.87 | 10.63 | -17.86 | 11.12 | 0.75 | 22.18 |
|  | mono non-forested micro+macro | 14.72 | 5.44 | 23.67 | 8.58 | -23.98 | 7.90 | 25.62 | 31.77 |
|  | mix non-forested micro+macro | 14.49 | 6.04 | 27.73 | 12.71 | -16.46 | 7.05 | 21.05 | 25.13 |
|  | mono forested micro | 10.62 | 3.92 | 13.25 | 6.27 | -19.35 | 11.32 | 7.47 | 13.67 |
|  | mix forested micro | 8.96 | 5.55 | 15.56 | 6.02 | -14.10 | 12.59 | 4.49 | 16.41 |
|  | mono forested micro+macro | 22.87 | 14.34 | 36.85 | 20.37 | -20.25 | 15.11 | 40.53 | 28.11 |
|  | mix forested micro+macro | 21.60 | 16.51 | 36.23 | 18.20 | -15.72 | 16.58 | 35.33 | 28.64 |


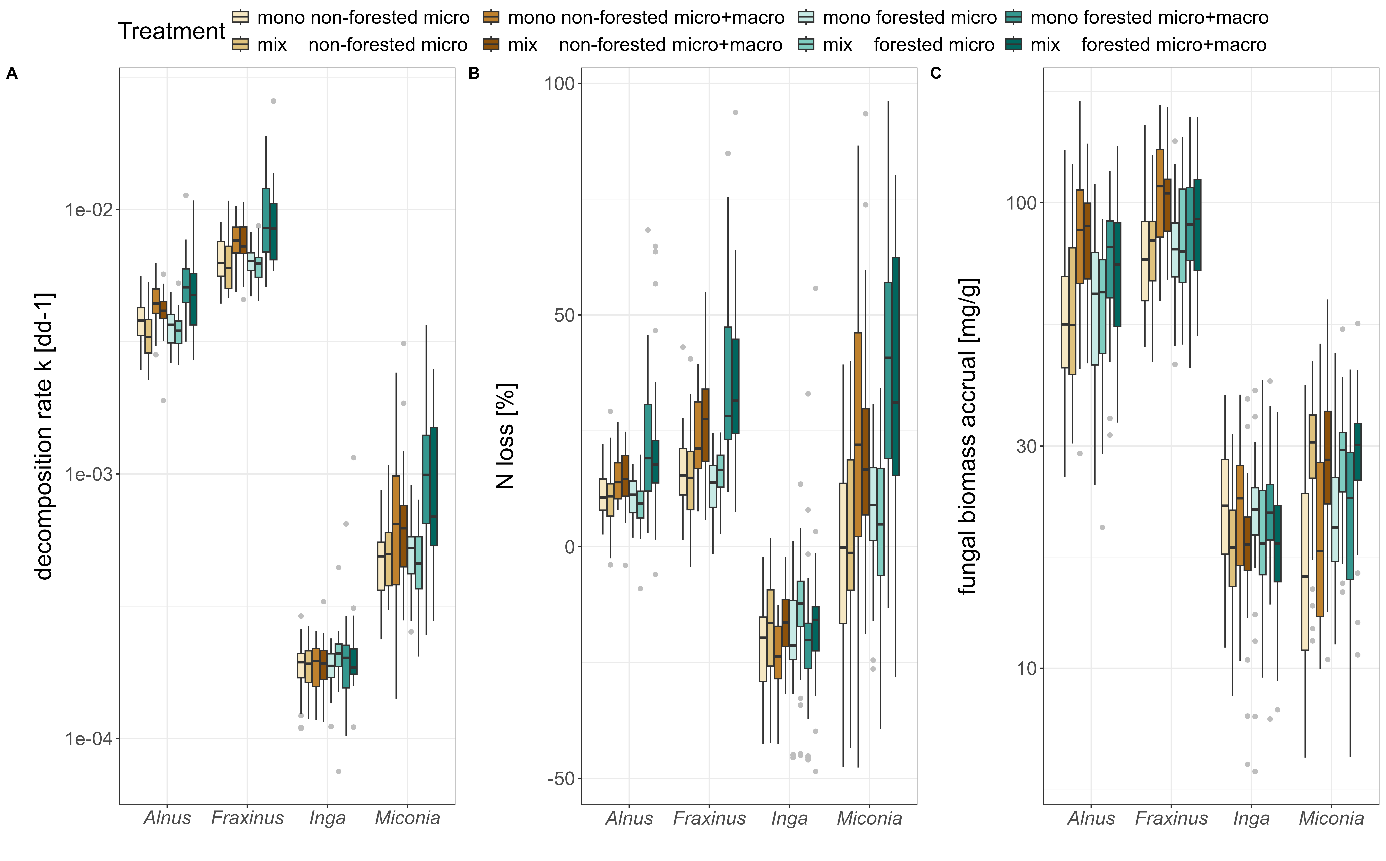


**Figure S1**: Untransformed values for each ecosystem function, treatment and across all four leaf species *Alnus*, *Fraxinus*, *Inga* and *Miconia* corresponding to Table S7.
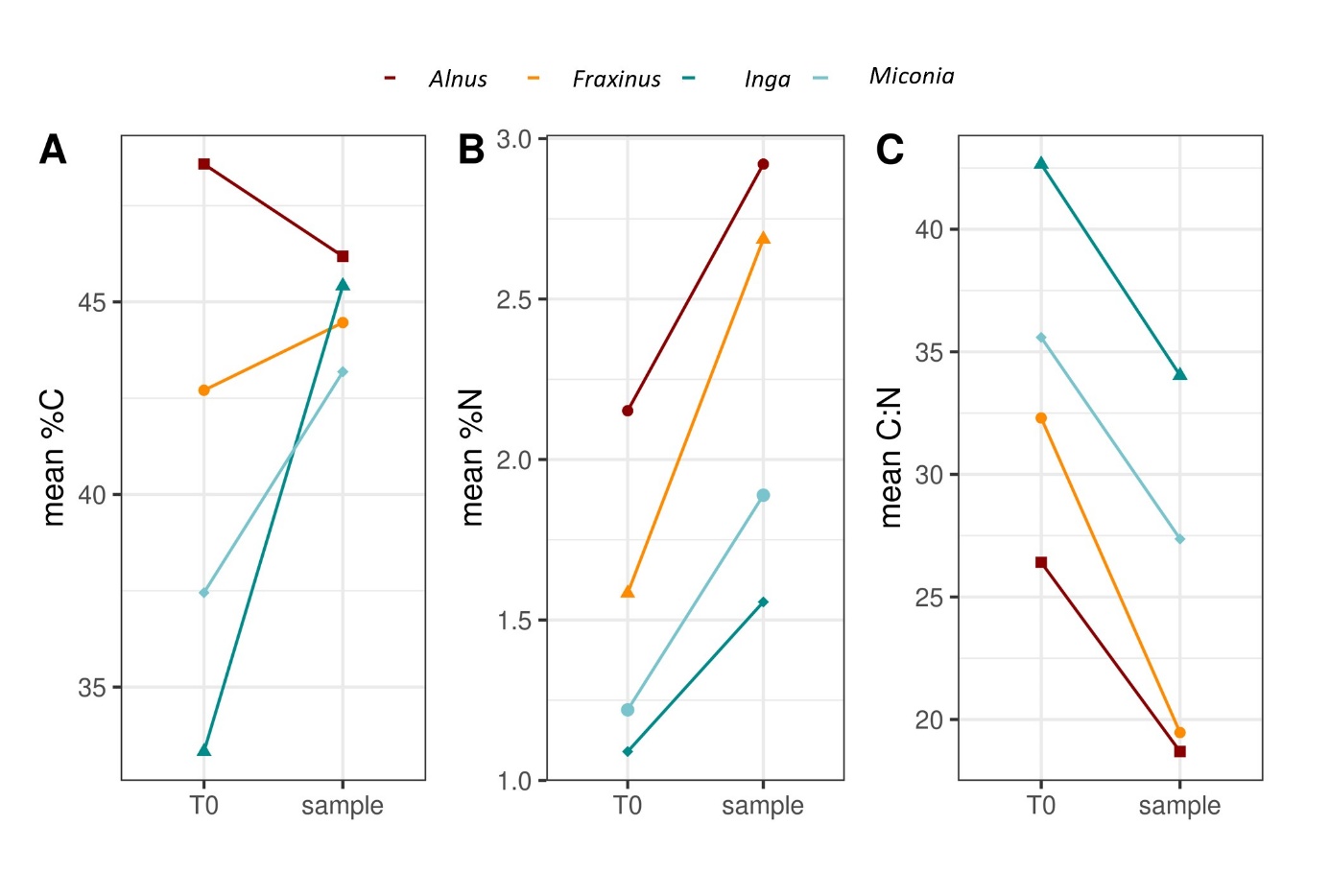


**Figure S2**: Mean initial and experimental %C, %N and molar C:N ratios of leaf litter for the four leaf species *Alnus*, *Fraxinus*, *Inga* and *Miconia*.


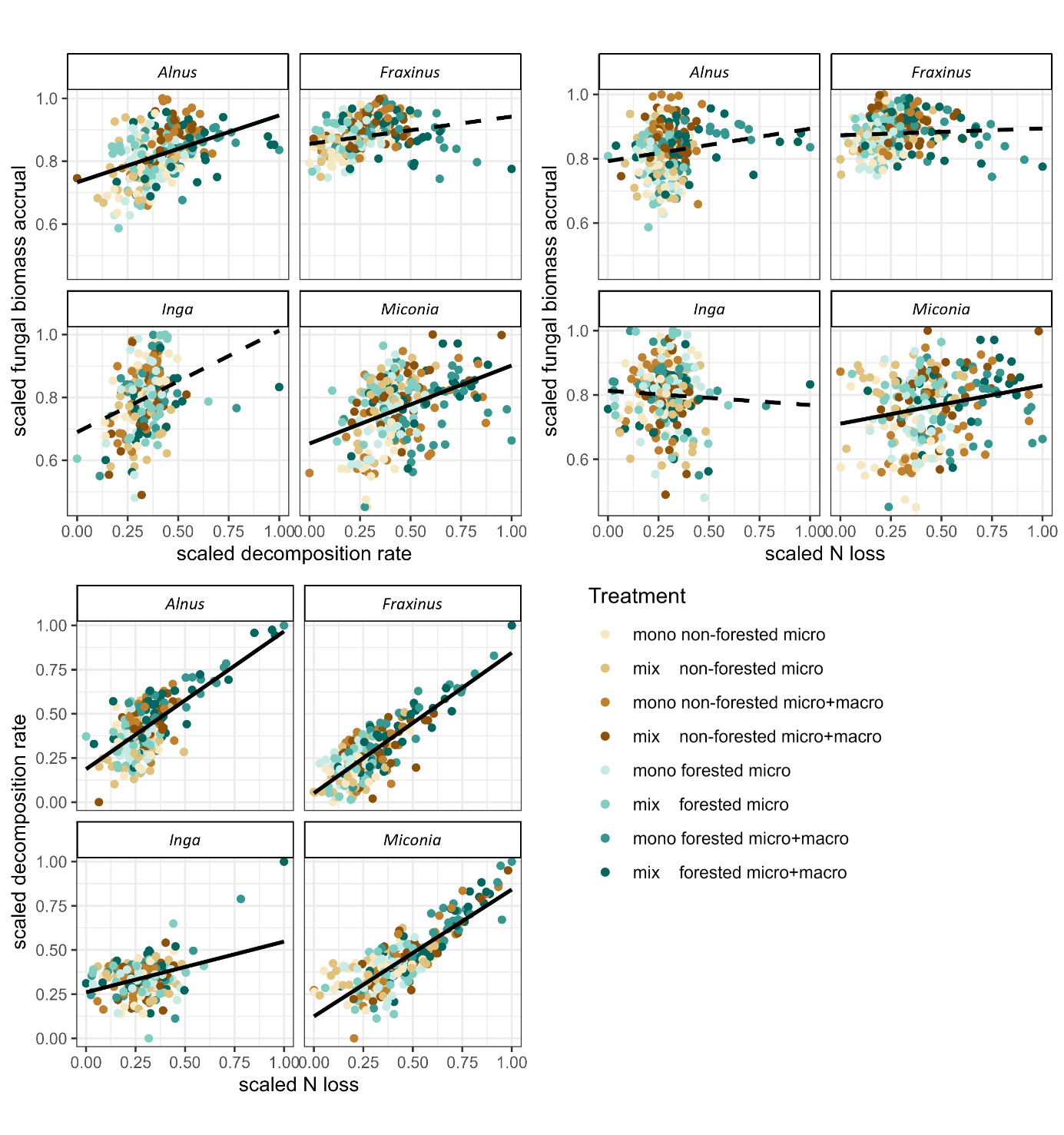


**Figure S3**: Relationships between ecosystem functions for the four leaf species. Solid lines indicate statistically significant slopes, and the colours of the points show the different treatment combinations. Details for each slope can be found in Table S5.
